# Dickkopf-Related Protein 1 Inhibits the WNT Signaling Pathway and Improves Pig Oocyte Maturation

**DOI:** 10.1371/journal.pone.0095114

**Published:** 2014-04-16

**Authors:** Lee D. Spate, Alana N. Brown, Bethany K. Redel, Kristin M. Whitworth, Clifton N. Murphy, Randall S. Prather

**Affiliations:** Division of Animal Science, Animal Science Research Center, Columbia, Missouri, United States of America; Institute of Zoology, Chinese Academy of Sciences, China

## Abstract

The ability to mature oocytes *in vitro* provides a tool for creating embryos by parthenogenesis, fertilization, and cloning. Unfortunately the quality of oocytes matured *in vitro* falls behind that of *in vivo* matured oocytes. To address this difference, transcriptional profiling by deep sequencing was conducted on pig oocytes that were either matured *in vitro* or *in vivo*. Alignment of over 18 million reads identified 1,316 transcripts that were differentially represented. One pathway that was overrepresented in the oocytes matured *in vitro* was for Wingless-type MMTV integration site (WNT) signaling. In an attempt to inhibit the WNT pathway, Dickkopf-related protein 1 was added to the *in vitro* maturation medium. Addition of Dickkopf-related protein 1 improved the percentage of oocytes that matured to the metaphase II stage, increased the number of nuclei in the resulting blastocyst stage embryos, and reduced the amount of disheveled segment polarity protein 1 protein in oocytes. It is concluded that transcriptional profiling is a powerful method for detecting differences between *in vitro* and *in vivo* matured oocytes, and that the WNT signaling pathway is important for proper oocyte maturation.

## Introduction

*In vitro* maturation (IVM) of mammalian oocytes has long been a problematic step for the *in vitro* production of embryos. While there have been major advances in improving both oocyte maturation and embryo culture over the past decades, and oocytes can successfully be matured *in vitro*, the rate of maturation and extent of maturation is lower than *in vivo* matured oocytes. Maturation of pig oocytes *in vitro* is even lower as compared to other livestock species [1], [2]. While IVM pig oocytes are capable of being fertilized, developing to the blastocyst stage and producing offspring after transfer to a surrogate, the developmental competence of these oocytes is lower than *in vivo* produced oocytes; partially due to a lower number of nuclei in the resulting blastocyst [3], implying that the quality of the blastocyst is lower. Incomplete oocyte maturation can cause abnormal chromosome segregation and reduce the developmental capacity of the embryos [4], [5]. While much of the focus trying to manipulate and understand culture conditions has been post-fertilization, the area for the greatest gain might be during IVM. By improving the oocyte's overall health *in vitro*, the stage could be set for improved development by providing the oocytes most of what they need to be developmentally competent.

To address the discrepancy of the quality of *in vitro* versus *in vivo* matured oocytes, we transcriptionally profiled IVM and *in vivo* matured oocytes by using next generation Illumina sequencing. One of the pathways that was overrepresented in the IVM oocytes was the Wingless-type MMTV integration site (WNT) pathway (see data below).

The WNT pathway and WNT related proteins are associated with embryogenesis, cancer, embryonic stem cells, and signal transduction. There are many members of the WNT signaling pathway that are expressed in ovaries, oocytes and early embryos of mice [6]. Indeed, WNT related proteins Frizzled Family Receptors (FZD1, FZD4, and FZD5) are present at the oocyte to embryo transition [6] and WNT ligands (WNT1, 3, 5A and 7A/B) are present in the cytoplasm of early mouse embryos. In addition, the WNT pathway plays a role in the fate of stem cells [7] as stimulation of this pathway acts as stem cell growth factors [8]. Furthermore, WNT pathway components are responsible for meiotic spindle orientation in *Caenorhabditis elegans* [9]. Inhibition of the WNT signaling pathway during early embryo development is deleterious in the cow [10], but not the mouse [11]. Interestingly, Dickkopf-related protein 3 (DKK3: thought to be a negative regulator of the WNT signaling pathway) is present in the oocyte and early mouse embryo [6], but it is controversial as to whether or not it is directly involved in the WNT pathway [12], [13]. Since the FZD1 receptor is located on the mouse oocyte as it matures, WNT ligands may control gene expression through a WNT-initiated signaling cascade during oocyte maturation [6]. Harwood et al [6] maintain that all the molecules needed for activation of alternative WNT pathways exist in the growing oocyte; therefore, repression of WNT and regulation of WNT related gene expression can be controlled by cross-talk between the oocyte and granulosa cells to aid in the maturation of the oocyte. Little more is known about WNT pathways during oocyte growth and maturation. We propose that WNT ligands might play a role in regulating oocyte maturation in the pig. Here we characterize the response to inhibiting the WNT pathway by adding Dickkopf-related protein 1 (DKK1) to the maturation system.

## Results

### Illumina sequencing of MII oocytes produced through IVM or in vivo

*In vitro* and *in vivo* produced metaphase II oocytes were analyzed by Illumina deep sequencing that generated 100 bp reads. The reads were aligned to our porcine custom database [14], yielding 18,141,082 reads aligning to the genome. Construction of a 95% confidence interval determined that a mean number of 7 reads or more were needed to be statistically greater than that of a mean of 0. A search for aromatase (*CYP19A1*), a transcript that is highly abundant in cumulus cells, was made to validate that there was no cumulus cell contamination. The mean number of normalized reads was 0.53 for the IVV oocytes and 0.08 for the IVM oocytes. The inability to detect more than 7 reads implies that there was little, if any, contamination by the cumulus cells. Those alignments with at least 7 reads, a p value of 0.1 (Students *t*-test) or lower, and at least 2 fold difference in abundance resulted in 1,316 transcripts that were more or less abundant in the two groups of oocytes. DAVID [15], [16] analysis showed a number of pathways that were over- or under-represented in the two groups (Table 1). After mining through this data we noted that there were several genes related to the WNT pathway that were over represented in IVM oocytes; specifically, *WNT7A*, *FZD4*, and *DVL1*. These are three of the early gene members in the WNT signaling pathway. We then decided to perturb this pathway during *in vitro* maturation and possibly effect oocyte maturation, subsequent embryo development, and embryo quality.

**Table 1:** David gene pathways that were mis-represented in the *in vitro* matured oocytes.

Gene Pathways That Were Less Represented in the *In Vitro* Matured Oocytes	
Term	P-Value
T cell receptor signaling pathway	0.002
Regulation of actin cytoskeleton	0.026
Progesterone-mediated oocyte maturation	0.029
Ubiquitin mediated proteolysis	0.037

Gene Pathways That Were More Represented in the *In Vitro* Matured Oocytes	
Term	P-Value
Spliceosome	0.0001
Cell cycle	0.0004
Melanogenesis	0.005
DNA replication	0.005
Ubiquitin mediated proteolysis	0.008
Vibrio cholera infection	0.012
Lysosome	0.016
Basal cell carcinoma	0.037
Pathways in cancer	0.029
*WNT* signaling pathway	0.040

### Real time validation for WNT7A, FZD4, and DVL1

**Figure 1:**
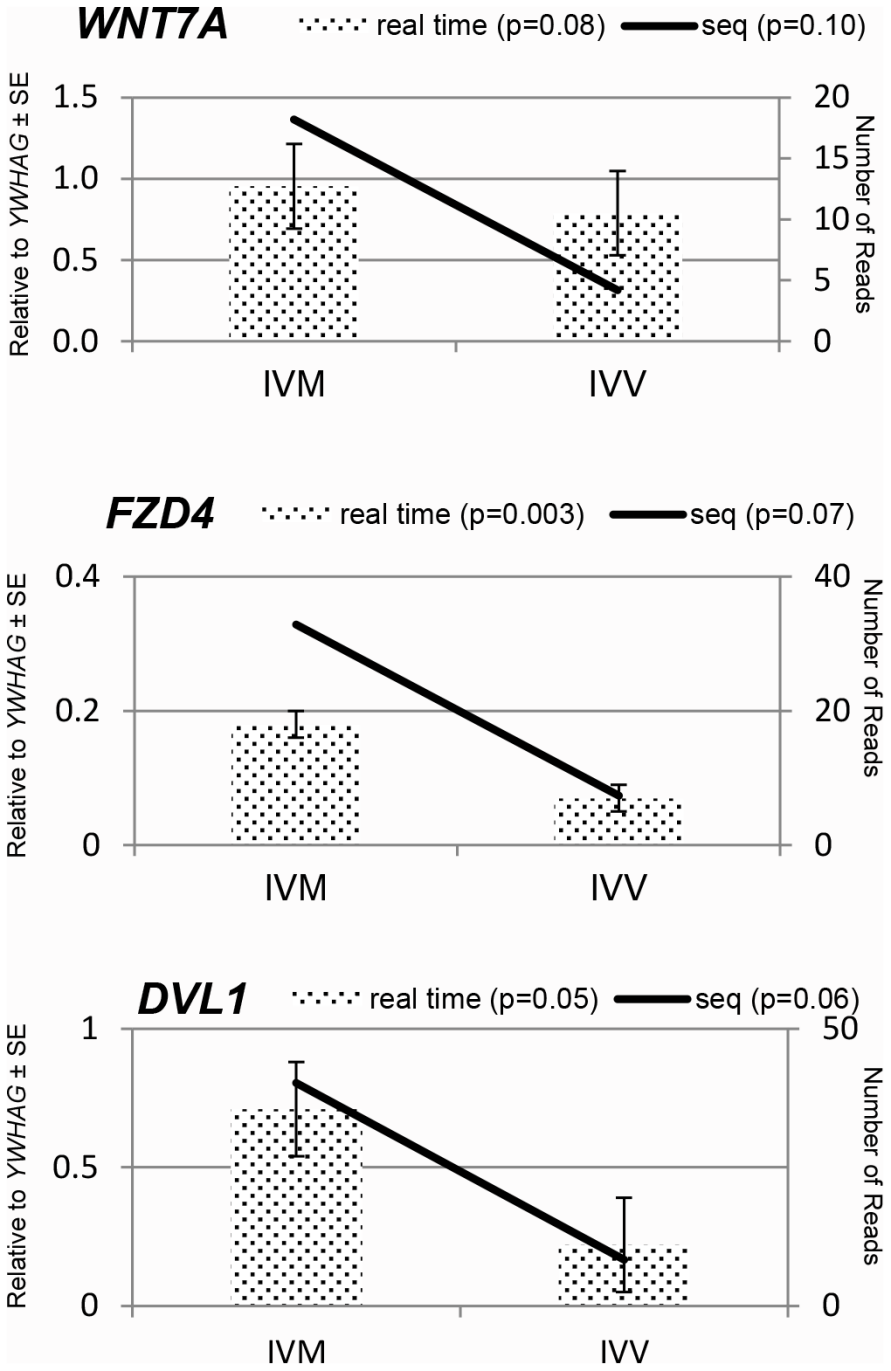
Real time PCR validation of Illumina sequencing data for *WNT7A*, *FZD4* and *DVL1*.

Real time PCR did validate the Illumina sequencing data with all three transcripts showing a higher abundance in the IVM oocytes (Fig. 1). The abundance of message for eight additional genes was determined. These genes were selected based on their similar or differential representation in the Illumina sequencing. Of the other three transcripts that appeared more abundant based on the Illumina sequencing, two (solute carrier family 7 (cationic amino acid transporter, y+ system), member 3 (*SLC7A3*) & zona pellucida protein 2 (*ZP2*)) were validated by real time PCR and the third (*SOX4*), while not different, showed the same directionality. Of the other five that were not significant by Illumina sequencing, four (heat shock protein 90 (*HSP90AA1*), mitochondrial ribosomal protein S36 (*MRPS36*), pyruvate dehydrogenase complex component X (*PDHX*), ubiquitin associated protein 2 (*UBAP2*)) were determined to be significant by real time PCR.

### Maturation of oocytes with DKK1

After maturing the oocytes with DKK1 there was a higher percentage of oocytes that developed to the metaphase II stage (Table 2). While there was no difference between the percentage cleavage (38.2% vs 35.0%) or the percentage of blastocysts (31.8% vs 27.8%), there was a significant difference in that DKK1 treated oocytes had more nuclei when they developed to the blastocyst stage compared to the controls (Table 2).

**Table 2:** Maturation of oocytes in the presence or absence of DKK1 (data represents four replications).

Treatment	Total	LS Means Percent Metaphase II ±SE, n	LS Means Percent Cleavage ±SE, n	LS Mean Percent Blastocysts ±SE, n	LS Mean Number of Nuclei in Blastocysts ± SE, n
CONTROL	661	73.1^a^±0.05, 483	35.0±2.1, 173	27.8±1.6, 145	28.6^a^±1.0, 67
DKK1	543	82.1^b^±0.06, 446	38.2±2.2, 166	31.8±1.6, 142	32.7^b^±0.9, 83

a,b Within a column P<0.05.

**Figure 2:**
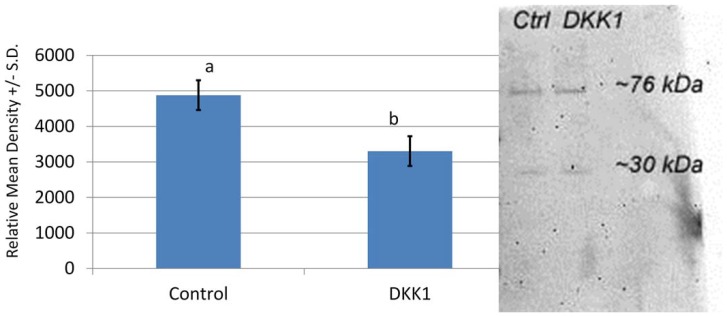
Western blot of DVL1 in oocytes matured in the presence or absence of DKK1. The mean densitometry of the two bands (30 and 76 kDa) was different between the two treatments ^a,b^p = 0.01.

We were unable to identify an antibody that recognized pig WNT7A or FZD4, so anti-DVL1 was custom made based on the pig sequence. Western blot analysis showed a decrease in DVL1 in response to culture with DKK1 (Fig. 2).

## Discussion

In a previous study evaluating transcript abundance in oocytes from cyclic versus prepubertal females, it was shown that development was lower from the prepubertal derived oocytes and about 10% of the transcriptome was different between the two groups of oocytes [17]. Message associated with metabolism and biological processes were higher in oocytes from cyclic females, while oocytes from prepubertal females had an increase in the abundance of message for translation [17]. A more targeted approach identified a number of transcripts related to apoptosis, fatty acid oxidation, and glycolysis that were higher in oocytes from prepubertal animals as compared to adult animals [18]. In contrast, many transcripts associated with redox homeostasis were lower in the prepubertal oocytes compared to the cyclic oocytes [19].

While the list of pathways that were identified as different between the *in vitro* and *in vivo* matured oocytes somewhat overlaps with the adult versus prepubertal comparison above, the list provides a number of unique directions to pursue; here we focused on the WNT pathway.

After a misrepresentation of the three WNT pathway related transcripts was discovered in our Illumina sequencing data, and subsequently validated via real-time PCR, we wanted to find a way to manipulate or correct the over representation. One such idea was to add DKK1 to the maturation medium. DKK1 is a member of the DKK family that competitively binds to LRP. When bound to LRP it prevents LRP from interacting with FZD4 which is needed along with Frizzled to bind to WNT; thus inhibiting the WNT pathway. When added to the maturation medium the abundance of DVL1 decreased. By manipulating WNT signaling pathway related transcripts by competitive inhibition it appears as though IVM oocytes are more like their *in vivo* produced counterparts. In addition, there was a clear increase in maturation percentage of oocytes that are matured in the presence of 200 mM DKK1 compared to control oocytes. Subsequent development of the DKK1 treated embryos to the blastocyst stage was not significant; however, the increase in total cell number lends to the belief that adding DKK1 may be encouraging the production of higher quality oocytes. While the increase in the number of nuclei represents an advancement, the number of nuclei in blastocysts produced *in vivo* is still far greater than the 30 or so nuclei reported here. Clearly, more work needs to be done to improve the quality of the oocyte maturation, fertilization, and embryo culture systems. The importance of the WNT signaling pathway during oocyte maturation appears to be similar in species ranging from zebrafish [20] to rhesus monkeys [21].

While our direction of inquiry was toward the canonical signaling pathway because of the change in abundance of transcripts, it is possible that DKK1 may activate a non-canonical pathway as well as the active planar cell polarity pathway; generally the result of activation of this pathway is a calcium release that could act in the oocyte. It is possible that a calcium signal could alter message or protein abundance during maturation. Since calcium signaling is integral to sperm signaling at fertilization, it is not clear if there would be an advantage to calcium release in the oocyte prior to fertilization.

It is of interest to note that the Illumina sequencing and the real time PCR apparently did not have the same sensitivity. For example, Illumina sequencing did not detect a difference between maturation conditions for *HSP90AA1*, *MRPS36*, *PHDX* or *UBAP2*, while real time PCR detected a difference (P<0.09) for each message. While this observation may appear incongruous, a more serious concern would be raised if both methods detected a difference and those differences were in a different direction, e.g. the abundance was higher via real time PCR and lower in the Illumina sequencing dataset.

Another pathway that was over-represented in both the *in vitro* and *in vivo* matured oocytes was for ubiquitin mediated proteolysis. At first glance this observation appears incongruent. However closer examination shows that different transcripts are represented in each pathway. For example, both protein inhibitor of activated STAT3 (*PIAS3*) and *PIAS4* are represented as part of the ubiquitin mediated proteolysis and both are higher in IVM oocytes, while *PIAS2* is higher in the *in vivo* matured oocytes. Since PIAS4 is also a repressor of WNT [22] it might imply that the IVM oocyte is attempting to compensate for inadequate culture conditions by up-regulating or stabilizing message for *PIAS4*. Similarly, other ubiquitin mediated proteolysis pathway members were differentially represented in the two groups of oocytes. These include ubiquitin-conjugating enzymes (*UBE*) *UBE2J2*, *UBE2C*, *UBE2K*, and *UBE2O* which were higher in the IVM oocytes, while *UBE2D3*, *UBE2N*, and *UBE2W* were higher in the *in vivo* matured oocytes.

These data also show that global transcriptional profiling using Illumina deep sequencing data can be a useful tool to improve *in vitro* maturation. This was just one altered pathway from the sequence data set and just a few misrepresented transcripts. Thus, we believe this is only a start of what could be done using the sequence data to improve porcine oocyte maturation and *in vitro* development. With the DKK1 experiments we showed that perturbing the WNT pathway does make IVM oocytes behave more like *in vivo* matured oocytes. Although, another direction we propose to pursue would be trying to increase WNT representation during maturation and determine if that further harms oocyte and embryo development.

## Materials and Methods

### Animal Research

This study was carried out under the approval of the University of Missouri Animal Care and Use Committee (approval #6523). Euthanasia was carried out via injection of pentobarbital sodium and phenytoin sodium as approved by the American Veterinary Medical Association Panel on Euthanasia. The animals were treated humanely.

### Oocyte collection for Illumina sequencing of in vitro and in vivo matured oocytes

Oocytes were matured *in vitro* or *in vivo*. Three gilts were monitored twice daily for standing estrus. Two days after first detection of estrus, *in vivo* matured oocytes were collected immediately after euthanizing the gilts by flushing the oviducts with TL-HEPES supplemented with 0.1% polyvinyl alcohol [23]. The metaphase II oocytes were quickly collected into pools of 10 and snap frozen in liquid nitrogen for RNA isolation. The IVM oocytes were obtained by euthanizing three prepubertal gilts, from a similar genetic background, and recovering the ovaries. Follicles 2–8 mm in diameter were aspirated by using an 18 gauge needle and syringe. Oocytes with multiple layers of cumulus cells and uniform cytoplasm were placed in M199 supplemented with LH, FSH, and EGF for 42 hours [24]. Upon maturation, cumulus cells were stripped and healthy metaphase II oocytes were collected in pools of 10 from each female and snap frozen. Total RNA was extracted from three pools of 10 oocytes for both treatments with an AllPrep DNA/RNA micro isolation kit (Qiagen). cDNA was synthesized using oligo (dT′) primed reverse transcriptase with Superscript III (Invitrogen, Grand Island, NY). Second strand cDNA was synthesized using DNA polymerase I and each sample was sequenced twice using an Illumina Genome Analyzer II. Data were deposited at NCBI (RNA-seq data for IVV (n = 3) and IVM (n = 3) samples were submitted to the NCBI Sequence Read Archive (SRA) and are available under accessions SAMN02640035, SAMN02640036, SAMN02640037, SAMN02640038, SAMN02640039, and SAMN02640040, respectively). All reads were aligned to a 34,433 member custom built porcine transcriptome [14]. If any reads aligned with more than one transcript, they were discarded from the analysis due to ambiguity. A normalization factor for each sample was computed to correct for variations between samples by taking the sample with the smallest number of alignable reads and then deriving a ratio using the total number of reads for each of the remaining samples. After normalization, the two runs for each sample were summed to get a representative number of aligned reads for each biological replicate. A mean for each transcript was calculated for the three biological replicates for each treatment group. A 95%-confidence interval was constructed around the mean number of reads based on the calculated pooled variance of the two sequencing runs to identify the average number of reads for each transcript that was statistically greater than zero. A Student's *t*-test and an added stringency of at least a 2-fold change between the means for each transcript was then used to determine if the means of the two groups for each transcript were statistically different. We then used DAVID (http://david.abcc.ncifcrf.gov/) to find pathways that were mis-represented in the IVM oocytes.

### Real time validation

Real-time PCR was conducted for 11 candidate genes and a housekeeping gene (Table 3), using IQ SYBR Green Supermix (Bio-Rad Laboratories, Hercules, CA) and the amplified cDNA from each biological replicate (diluted to 5 ng/µL) as template. Primers (Table 4) were designed by using Integrated DNA Technology (Coralville, Iowa) software, and real time PCR was completed in triplicate for every biological replicate on the MyiQTM Single-Color Real-Time PCR Detection System (Bio-Rad Laboratories) to verify the differential expression of the chosen transcripts between IVM oocytes and *in vivo* derived oocytes. Primer efficiency tests were completed for each primer set by generating a standard curve using 10 ng dilutions of our 5 ng/µL reference cDNA pool. Real-time PCR was run at each concentration (5 ng/µL, 0.5 ng/µL, 0.05 ng/µL) to validate each primer set. Expression levels for each mRNA transcript were calculated in the same manner as we reported previously [14]. The abundance of each transcript was calculated relative to the reference sample and the housekeeping gene, tyrosine 3-monooxygenase/tryptophan 5-monooxygenase activation protein, gamma polypeptide (*YWHAG*) [14], [25]. The reference sample used for *DVL1* contained four biological replicates of *in vivo* fertilized/*in vivo* cultured blastocysts and *in vivo* fertilized/*in vitro* cultured blastocysts that were pooled together [14]. The reference sample used for *FZD4* and *WNT7A* contained pools of reproductive and non-reproductive tissues across different stages of development [25]. Expression levels between treatments were determined using the comparative threshold cycle (CT) method for each gene. The 2^-ΔΔCT^ values were first analyzed for normality and skewness. The resulting values were then analyzed using the general linear model (PROC GLM) in the Statistical Analysis System (SAS; SAS Institute, Cary, NC). Differences in expression were found by using the Least Squares Means (LSMeans) generated by PROC GLM (P<0.05) [26].

**Table 3:** Transcript abundance of selected genes as determined by Illumina sequencing and real time PCR.

	Illumina Sequencing			Real Time PCR		
Gene	*In Vivo* Matured # Reads ± SE	*In Vitro* Matured # Reads ± SE	P-Value	*In Vivo* Matured Relative Amount ± SE	*In Vitro* Matured Relative Amount ± SE	P-Value
*ATG4*	7.8	17.0	NS	3.2±0.5	3.3±0.5	NS
*HSP90AA1*	4.5	13.9	NS	7.7±0.6	5.8±0.6	<0.06
*MRPS36*	43.7	23.8	NS	3.85±0.4	0.7±0.4	<0.001
*PDHX*	99.8	69.1	NS	7.9±1.1	4.2±1.1	<0.03
*SLC7A3*	15.7	43.6	<0.02	1.8±0.4	3.6±0.5	<0.02
*SOX4*	62.2	21.6	<0.06	4.7±0.2	4.2±0.2	NS
*UBAP2*	26.5	86.0	NS	5.3±0.5	3.8±0.6	<0.09
*ZP2*	141.2	1,134.2	<0.06	84.1±16.8	272.0±16.8	<0.001

**Table 4:** Accession number and primer sequences used for transcript amplification.

Transcript Annotation	Accession #	Sense Primer	Anti-Sense Primer
*DVL1*	XM_003127500	TGGCTCAAGATCACCATCGCCAAT	AAGACGTAGTAGCACTGCTCGGAGA
*FZD4*	NM_001243330	CAGCACTCAGGCCAACGCCAT	AGAGCAGATCCGGGCTGCAGT
*WNT7A*	XM_003132396	AGCGATGCTGAGCTTGTCTTGTCT	TATTGCTATGTTGAGGCCCAGGGT
*YWHAG*	NM_012479	TCCATCACTGAGGAAAACTGCTAA	TTTTTCCAACTCCGTGTTTCTCTA
*ZP2*	NM_003460.1	TCCTGTGACCTGTAATGCCACACA	AACCTCAGGCCGTTTGTTGTTTCC
*HSP90AA1*	NM_005348.3	ACTCCGGGAAAGAGCTGCACATTA	AGACTTGGCGATCGTACCAAGGTT
*UBAP2*	NM_018449.2	GGCCAAGCCCTTGTCTTCACAAAT	AAATCCTGAGCCAAGGACGGATGA
*SLC7A3*	NM_032803.5	GCAGCGTTCATGGCATTCCTCTTT	ACACAAACAGCCACCAGGGAGTAA
*MRPS36*	NM_033281.5	AGGGTCGTTCAGGTAGTCAAACCA	TTGTTGACCCTGATACATCAGCAA
*PDHX*	NM_001135024.1	TGGTATCACAGCACGCAGTCTCTT	AGCACTCACAGCTTCACCTTCCTT
*SOX4*	NM_003107.2	AGGGCGGCTGGTTAATATCTCACA	AAGGATGGATACTGGTGGCAGGTT
*ATG4A*	XR_113562.1	CTTCTCCTACACCCATTTGTGCCA	TGACAGCTGGAGAATGGAGTCAGT

### In Vitro Maturation and DKK1 treatment

Ovaries were obtained from a Farmland, Incorporated slaughterhouse in Milan, MO and transported to the laboratory at ambient temperature. Ovaries were washed with physiological saline with 10 µg/mL gentamicin at 37°C. Follicles between 2–8 mm were aspirated using a 10 mL syringe and an 18 gauge needle. The aspirate then was washed in TL- HEPES [23] three times and oocytes with multiple layers of cumulus cells and homogeneous cytoplasm were then placed into maturation media either with or without 200 ng/mL DKK1 as recommended by the manufacturer (PeproTech 120–30). Cumulus cells were stripped after 42 hours by vortexing in 1 mg/mL hyaluronidase for 4 min. The cumulus cell-free oocytes were selected for the presence of an extruded polar body, thus considered to be at metaphase II, and data was collected for both treatments and recorded.

### In Vitro Fertilization and Embryo Culture

Metaphase II oocytes were washed in Modified Tris Buffered Media (MTBM) [24] and co-incubated in 100 µL of MTBM with 0.25×10^6^ frozen-thawed sperm for 5 hours [24]; this was considered Day 0. Five hours after fertilization the presumptive zygotes were washed in Porcine Zygote Media 3 (PZM3: [27]), transferred to 4 well Nunc plates and then cultured in 5% CO_2_ at 38.5°C for 28–30 hours, at which point cleavage data was obtained. The embryos were cultured in 5% O_2_, 5% CO_2_, 90% N_2_ at 38.5°C until day 6 at a density of 35–43 per 500 µL under an oil overlay. Development to blastocyst was recorded and total cell number was determined after staining with Hoechst 33342 (0.6 µg/mL) and visualized under UV light.

### Western blots of oocytes for DVL1

Fifty MII oocytes from either control or DKK1 treatments were collected washed in diethyl pyrocarbonate treated PBS with polyvinyl alcohol, snap frozen and stored at −80°C until ready for use in a western blot. The samples were thawed on ice, at which time 10 µL of 6x Laemmli's loading buffer (Boston Bioproducts, Ashland, MA. BP-110R) was added to each sample. The samples were then heated to 96°C for 5 minutes, centrifuged and run on a mini protein gel 4–20% (BioRad 4561093s). Proteins were then transferred to polyvinylidene fluoride membrane via an iBlot transfer apparatus (Invitrogen) and iBlot gel transfer stacks (Invitrogen). The membrane was blocked for 4 hours in tris buffered saline StarlingBlock blocking buffer (Thermo 37542) with 0.1% tween on a rocker. The membrane was then washed one time in tris buffered saline (Fisher BP2471-1) with 0.1% tween. Goat polyclonal primary antibody to DVL1 (custom made by Everest Biotech EB11466 by using peptide KLPVAPERVTLAD) was added in 1∶500 dilution and incubated overnight at 4°C. It was washed 5 times then placed in 1∶20,000 dilution of horseradish peroxidase conjugated rabbit anti goat secondary antibody (Pierce Scientific 31402) for 1.5 hours, washed again 5 times and then a 1∶1 mix of supersignal peroxide solution and Luminol/enhancer solution was added to cover the membrane for 3 minutes. The excess was drained off and the membrane was visualized on a Fotodyne imagining system (Highland, WI, USA) with a 5 min exposure. Later, the densitometry of the blots was analyzed using Foto/Analyst and total lab quant ID western blot analysis. The densitometry readings of both bands were summed together for the calculations. Three replications were completed and a paired one-tailed Student's *t*-test was used to detect differences.
